# Challenges and opportunities in uncertainty quantification for healthcare and biological systems

**DOI:** 10.1098/rsta.2024.0232

**Published:** 2025-03-13

**Authors:** Louise M. Kimpton, L. Mihaela Paun, Mitchel J. Colebank, Victoria Volodina

**Affiliations:** ^1^ Department of Mathematics and Statistics, University of Exeter, Exeter, UK; ^2^ School of Mathematics and Statistics, University of Glasgow, Glasgow, UK; ^3^ School of Mathematical Sciences, University of Southampton, Southampton, UK; ^4^ Department of Biomedical Engineering, Edwards Lifesciences Foundation Cardiovascular Innovation and Research Center, University of California, Irvine, CA, USA; ^5^ Department of Mathematics, University of South Carolina, Columbia, SC, USA

**Keywords:** uncertainty quantification, biology and healthcare, clinical decision support, mechanistic models, digital twins

## Abstract

Uncertainty quantification (UQ) is an essential aspect of computational modelling and statistical prediction. Multiple applications, including geophysics, climate science and aerospace engineering, incorporate UQ in the development and translation of new technologies. In contrast, the application of UQ to biological and healthcare models is understudied and suffers from several critical knowledge gaps. In an era of personalized medicine, patient-specific modelling, and *digital twins*, a lack of UQ understanding and appropriate implementation of UQ methodology limits the success of modelling and simulation in a clinical setting. The main contribution of our review article is to emphasize the importance and current deficiencies of UQ in the development of computational frameworks for healthcare and biological systems. As the introduction to the special issue on this topic, we provide an overview of UQ methodologies, their applications in non-biological and biological systems and the current gaps and opportunities for UQ development, as later highlighted by authors publishing in the special issue.

This article is part of the theme issue ‘Uncertainty quantification for healthcare and biological systems (Part 1)’.

## 1. Motivation

Computer modelling, simulation and data analysis are recognized tools for understanding our complex physical world. These tools are used in applications such as climate science [1–3] and aerospace [4], though their application in biological and healthcare systems is still in its infancy [5–7]. The COVID-19 pandemic highlighted the benefits, and shortcomings, of predictive modelling in biological and healthcare applications [8]. Numerous computational models quantified and predicted outcomes during the pandemic; however, modelling efforts were not entirely successful, in part owing to the novel strategies for data collection and the unprecedented speed at which the virus spread. In addition, simulation-based decision-making was cross-examined by the general public, as many research articles (in some cases up to 94% of all COVID modelling studies [9]) lacked some sort of confidence or credible interval assessment in the model predictions for the pandemic response. As noted by Jit *et al*. and James *et al*. [8,10], these efforts caused some individuals and groups to cast doubt on the role of pandemic modelling and simulation for planning purposes. This intersection of mathematical modelling, epidemiological expertise, statistical analysis and policy implementation was a spotlight on the topic of UQ within biology and healthcare.

There are fewer applications of UQ in biological and healthcare models for several reasons. First, models describing biological or healthcare-related concepts often lack foundational model infrastructure, such as momentum balance or energy conservation laws commonly used in physics. As a consequence, and as discussed later, models in biology and healthcare are highly susceptible to model misspecification [11,12], and handling of this model uncertainty typically requires sophisticated UQ techniques. A second reason is the heterogeneity of healthcare and biological data [13]. In particular, integrating data of different modalities, e.g. electronic health records, imaging data, as well as clinical data into the UQ framework, is a challenging task, and thus performing holistic UQ to account for as many sources of uncertainty as possible has not been pursued so far. Last, much of the research work in the mechanistic, biological modelling field is typically carried out in the absence of statistical experts (e.g. only in teams of engineers, mathematicians and domain scientists). Although most of these studies are typically focused on developing the models themselves, the lack of statistical input again limits the depth of UQ that may be available for non-statistical (or non-UQ) experts.

Inspired by this, a Lorentz Center workshop on ‘Uncertainty Quantification for Healthcare and Biological Systems’ was held in April 2023. A group of junior, mid-career and senior scientists across biology, statistics, mathematics, engineering and computer science met to discuss the importance, implementation, gaps and opportunities for UQ in healthcare and biological models. The major outcome of this workshop included a collection of challenges for UQ in biological and healthcare applications, with limitations in communication between modelling and simulation experts, statisticians and domain scientists being at the forefront. To promote UQ in this sector, we have constructed a special issue collection of papers focused on this critical area.

In this introduction to the special issue, we provide an overview of computational modelling and simulation in healthcare and biology, and the key gaps in UQ within this field. We introduce modelling and simulation in established fields (e.g. engineering and physical sciences), as well as newer healthcare applications, and provide definitions and concepts in the field of UQ. Next, we present an extensive summary of gaps and opportunities in UQ in biology and healthcare modelling. We then introduce the special issue, which includes a collection of articles at the interface of modelling and simulation, healthcare and biology, data integration and machine learning (ML), and UQ.

## 2. Modelling, Simulation and *Digital Twins*

Mathematical and computational models provide an abstract representation of physical systems of interest, enabling advanced analysis, simulation, optimization and prediction. This leads to significant improvements in performance and efficiency in domain applications. Throughout the main text, we refer to these first-principal-based models as **mechanistic models** [14,15].

With the increase in computer power and the availability of data, terms such as mathematical modelling, simulation and digital twins are becoming increasingly common. Although in many applications they are used interchangeably, there are still communities that have their own precise definitions (e.g. [16]). While modelling creates mathematical representations of a system, simulation refers to applying this model to conduct numerical experiments and analyse results. Simulation uses computational methods to solve model equations and visualize results. We define a **digital twin** to be a dynamic, virtual replica of a physical system that can be continuously updated with new data, allowing up-to-date monitoring, analysis and optimization of the system. A digital twin can run any number of useful simulations to study multiple processes and is linked to physical data sources so that updates reflect the current state of the physical object [17]. Simulations usually do not require real-time data, but digital twins are ideally designed around a two-way flow of information. This is the main drive for the increased use of digital twins, particularly in healthcare applications [18].

### (a) Application and value in engineering and physical sciences

In engineering and physical sciences, mathematical models are typically formulated using systems of equations that define the relationships between variables and dynamics based on physical laws. They are powerful analytical tools that help us better understand physical phenomena and simulate complex systems. For instance, the Lotka–Volterra (predator–prey) model [19] in ecology is used to study the interactions between predator and prey species over time. In addition, researchers employ mathematical models to explore different scenarios and their potential effects. For example, four forcing scenarios, representative concentration pathways [20], are used in climate models to study their effects on global climate systems.

Mathematical modelling and simulation are increasingly used as decision support tools. Modern engineering uses computer modelling to develop designs in a cost-effective way [21]. For instance, the Langley grid-back booster for rockets at NASA was developed through the use of computer simulators [22]. In geology, computational models are employed for hazard control with geophysical flow models to assess hazard threats owing to volcanic avalanches [23]. In climate research, the Coupled Model Intercomparison Project (CMIP6) brings together climate modelling centres to produce multi-model climate projections to explore a number of science and policy questions [24] that provide ‘evidence base’ for policy decisions globally [25].

Decision-making can be further enhanced by utilizing digital twins. The concept of a digital twin [1] originated in the early 2000s in manufacturing and was formally introduced by NASA in aerospace engineering [26] where they were used to mirror the behaviour of aircraft engines and other critical system components in space in real time. Today, digital twins can predict engine failures in commercial aircrafts before they occur while also enhancing flight path updates through continuous system monitoring [27]. Other examples include engineering and industrial manufacturing, and smart city simulations [28]. Despite the success of digital twins in industrial sectors, it is still a relatively new concept in environmental sciences with ambitious projects such as Destination Earth (DestinE) starting to appear to support climate change adaptation [29]. Dale *et al*. [1] outlined the main challenges in adopting digital twins, which include modelling complex environmental systems and environmental information integration.

Advances in computational power have introduced new challenges in modelling and simulation. While they enable more accurate and complex simulators, these models are often computationally demanding, thus increasing computational costs. Multi-fidelity computer models [30–32] address this by combining computationally inexpensive models with fewer runs of expensive high-fidelity models. Multi-scale models are also used to integrate simulations at different scales. For example, aerospace models are built to test the safety of aircraft materials [27]. To reduce costs, a variety of length scales ranging from small sections of carbon fibre-reinforced plastic, to aeroplane wings, to the full aircraft are tested.

### (b) Application to biology and healthcare

Biological systems are distinct from physical systems in numerous ways, one of which is the relatively fewer number of ‘laws’ that govern biology. Laws of physics, motion and momentum can be applied in biological systems, but these laws only cover a few applications (e.g. biomechanics, biofluids). Thus, biological and healthcare models are derived in a way that is specific to the population, organism or biological process of interest.

Biological and healthcare simulations have recently made it into the public limelight. A food and drug administration-approved tool for insulin delivery is based, in part, on mechanistic modelling (using ordinary differential equations (ODEs)) [33]. Simulations of heart biomechanics, heart valve function and blood flow have all contributed to new technologies in the healthcare industry [15]. HeartFlow© is an image-based simulation platform that combines medical imaging, computational haemodynamics and ML to predict coronary artery disease risk factors [34]. More resources are now dedicated towards modelling for medical device development [35], ‘patient-specific’ models [36] and *in silico* clinical trails [37].

The use, and criticism, of biological modelling was highlighted during the COVID-19 pandemic [38–40]. The European Union (EU) used the ‘CovidSim’ software, developed in part by Neil Ferguson at Imperial College, London [41], for pandemic response. Though the model fared well in helping plan effective mitigation strategies, a study by Edeling *et al*. [42] quantified the uncertainty and biases in the CovidSim model. CovidSim includes over 900 model parameters, of which 60 were included in the UQ analysis by Edeling *et al*. The authors found that 19 of the 60 parameters were influential on the model outputs and provided nearly 300% variability in the model outputs, yet uncertainty accredited to model misspecification and variability in the simulation settings were not investigated.

One of the most exciting, cutting-edge areas of computational biology and healthcare is in the development of human digital twins. This is part of the broader goal of the Virtual Physiological Human’s [43] EDITH project in the EU. Funded by the European Commission, EDITH aims to develop a virtual human twin of human physiology in an attempt to better address pathological conditions [44]. Within this road map is the idea of model ‘credibility’, which includes aspects of UQ. A workshop hosted by the National Academies of Science, Engineering and Medicine in the USA identified the gaps and future directions in the development of digital twins [17]. A key development of this workshop was updated definitions for digital twin technologies, including (i) the ability of a digital twin to have predictive capabilities and (ii) the need for bidirectional interactions, such that the digital representation of the physical system can be updated with new data and information. In documenting the current gaps and unmet needs in digital twin technologies, the committee noted that the area of verification, validation, and UQ (VV&UQ) was especially important in constructing physiological digital twin technologies [17,18].

The report [17] also made an important distinction between what are now new ‘digital twins’ and the previous ideas surrounding patient-specific models. The bidirectional nature of the digital twin implies that the model and data are not single instances, but rather *continuously updated*. A notable example is the recent study by Chadhuri *et al*. [45], who provided a computational oncology digital twin that was calibrated over multiple time points and provided output uncertainty over an extended period of time. As digital twins become reality, a next step will be in clinical decision support using these twins. This idea is echoed by Huberts *et al*. [46] in the cardiovascular modelling space, whereas the article by Sesen *et al*. [47] considered a purely data-driven Bayesian network approach to decision support in lung cancer. Quantifying uncertainties will be paramount as these techniques and strategies are implemented in clinical decision-making.

There are several examples and outlines of digital twins in specific domains, including cardiology [15,18], pulmonary hypertension [48], oncology [45] and immunology [49–51]. In addition, some initial pipelines for UQ in biological and healthcare systems have been proposed [14,52]. Though UQ is also being applied in the context of ‘model-free’ artificial intelligence (AI) and ML methodologies, this is outside the scope of the present review (see Seoni *et al*. [53] for details).

## 3. Uncertainty Quantification (UQ)

Mathematical models and digital twins are not perfect replicates of the real-world systems they represent. To support high-stake decisions using computer-based simulations, we need to ensure that predictions produced by these models are credible. The study in [54] identified three key principles to assess model credibility: verification, validation and uncertainty quantification. Verification ensures that the numerical implementation and solution of the model are correct. Validation tests whether the model itself sufficiently captures the dynamics specific to the quantity of interest (sometimes called the ‘context of use’). The last ingredient, UQ, consists of formally identifying and quantifying uncertainties within the model and associated with the data when performing model-based inference. Although UQ is a well-established and widely recognized discipline within the engineering and physical sciences communities, UQ for healthcare and biological systems is under-developed due in part to the complexity of biological and healthcare data, as well as the greater amount of ‘unknowns’ in the development of biological models.

One of the main aims of this special issue is to improve modellers’ and healthcare and biological researchers’ understanding and comprehension of UQ, which can significantly encourage the adoption of UQ as part of the credibility assessment. Many UQ ideas have been echoed in certain healthcare domains, more specifically in the cardiovascular modelling community and the systems biology domain. These ideas were presented in the ‘fickle heart’ meetings [55], as well as the ‘Special Issue on Uncertainty Quantification, Machine Learning, and Data-Driven Modeling of Biological Systems’ [56]. We also note that the field of *in silico* clinical trials has adopted multiple ideas from the UQ field [57], including the Comprehensive *in vitro* Proarrhythmia Assay (CiPA) initiative [58]. The goal of CiPA is to reduce the chance of cardiac toxicity in clinical trials, and has provided a roadmap for how UQ can be used in guiding medical and pharmaceutical directions.

**Figure 1.**
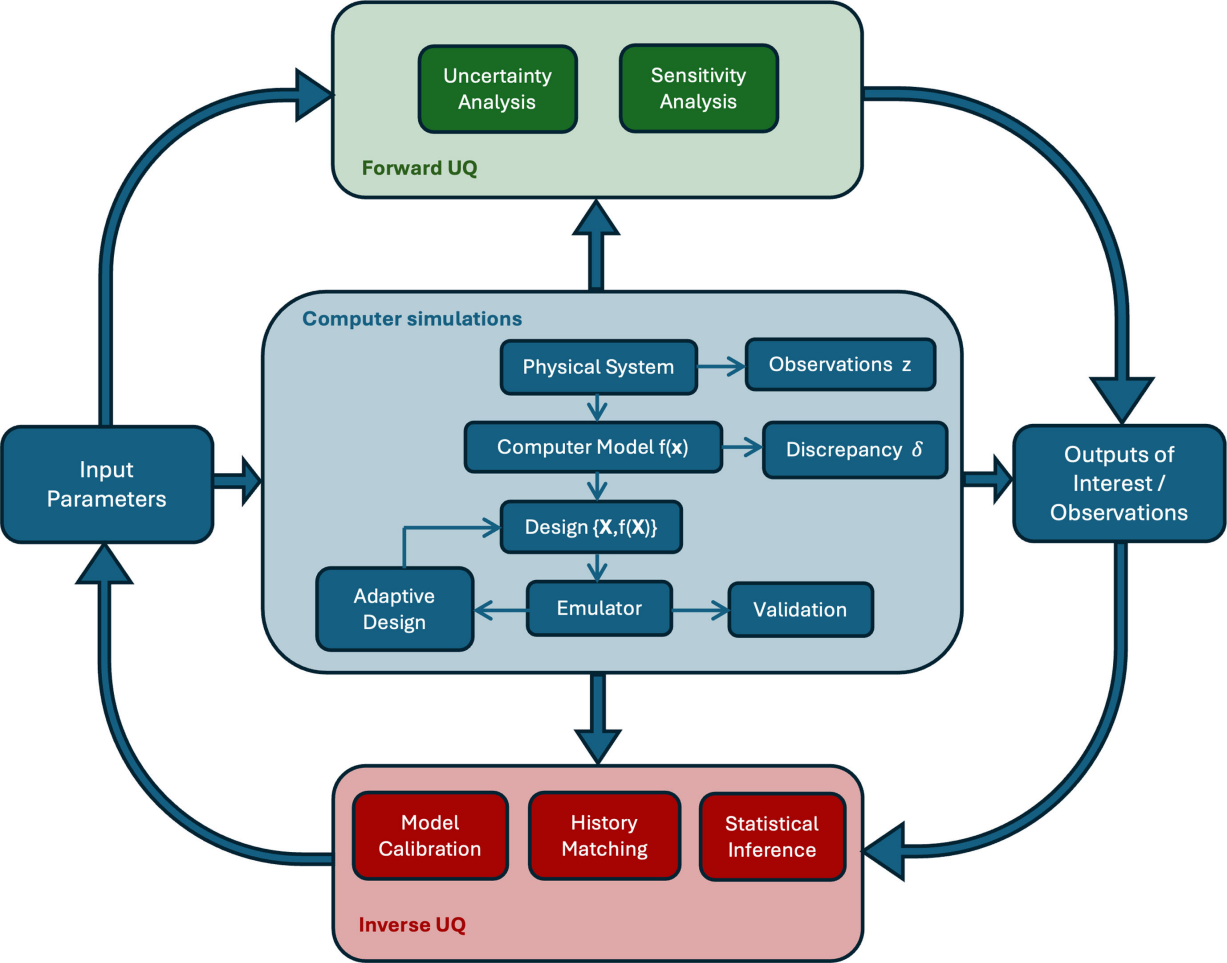
UQ pipeline. At the center of the UQ pipeline is the computer model f that provides a virtual representation of the physical system. Each coloured box corresponds to a distinct branch of UQ methods described in §3. The flowchart demonstrates the iterative nature of UQ, where input parameters x drive simulations that are continuously refined and validated, linking model outputs to real-world observations (z) from the physical system.

Figure 1 demonstrates a UQ pipeline, including some essential components and their connections. In particular, we consider a computer model, a mathematical (coded) representation of a physical process of interest, expressed through a mathematical function f that takes varying input parameter values x and returns output f(x). Observations z are collected from the physical system to aid in the design and calibration of the computer model. We recognize that the computer model is not a perfect representation of the physical system and therefore include the model discrepancy, denoted as δ, in our analysis. As part of the UQ pipeline, we consider both forward and inverse UQ. Forward UQ, typically performed first, involves propagating uncertainties in model inputs through the mathematical model to determine how they affect the outputs. Inverse UQ, typically performed second, works in reverse; it quantifies the uncertainties in the inputs based on observed data, z, or desired outcomes. Performing forward and inverse UQ analyses typically require numerous computer model runs. However, limitations arise when simulators have an extensive infrastructure, leading to high computational costs and thus restricting the number of available simulations. To avoid this computational burden, it is common to construct a surrogate model (an emulator) that provides a fast approximation of the computer model output. In the remainder of this section, we discuss UQ methods shown in figure 1 in detail.

### (a) Computer simulations and emulators

When dealing with expensive simulation models, a solution is to adopt an emulator, which is a computationally fast approximation to the model. An emulator requires an input training design $X=(x_{1},x_{2},\ldots ,x_{n})$, and the corresponding model realisations $f(X)=(f(x_{1}),\ldots ,f(x_{n}))$. It is common to adopt ‘black-box’ approaches when constructing emulators, where the focus is on modelling the input–output relationships without needing to fully understand the complex components of the simulation model. Extensive research exists in the field of emulators and surrogate modelling with methods including Gaussian processes (GPs) [59–61], polynomial chaos [62,63] and neural networks [64,65]. There are also innovations in *physics-informed* emulation [64,66,67], where underlying mechanisms of the system (e.g. the model equations) are integrated with observations to improve the emulator’s interpretability and ensure that physical constraints of the system are preserved.

As shown in figure 1, after constructing an emulator, it is crucial to validate it to ensure an accurate representation of the model’s behaviour across the input space. An ill-fitting emulator can lead to misleading uncertainty estimates or predictions. The accuracy of an emulator is also greatly influenced by the design of the training points [68,69]. It is important to balance between a space-filling design and one that captures key features of the physical system (exploration versus exploitation) [70]. Optimal designs can be either single-shot, using the entire budget at once, or adaptive, selecting new parameter locations sequentially based on predefined criteria (see [71–75] for details). As shown in the pipeline, an initial design is generated (e.g. a Latin hypercube, Sobol’ sequence or sparse-grid design [75]), and an emulator is built. An adaptive design (e.g. mean-squared-prediction error or mutual information) then uses information from the emulator to optimally select new input locations.

As discussed in §2a, physical systems are often represented by complex mathematical models that can involve high-dimensional input and output spaces. To simplify the analysis of these models within the UQ framework, it is common to adopt projection-based reduced-order modelling (ROM) methods [76–78]. After performing ROM, it is then possible to construct surrogate models and perform an inverse problem using the reduced space [79,80]. In particular, studies in [78,80,81] used principal component analysis before emulation since it allows to compress the large output space into a smaller, more manageable set of principal components that capture the majority of the variation in the data. In applied mathematics, a similar approach is carried out using proper orthogonal decomposition [75]. In addition, as mentioned in §2a, highly complex computer models can be run at different levels of complexity or resolution. Emulators have been developed for both multi-level [82] and multi-scale models [83].

The output of, for example, engineering models is typically deterministic, where the output is identical if the model is run multiple times at the same parameter settings. Alternatively, there are many cases in healthcare and biology, such as epidemiology, where a random component causes the output to be stochastic. The additional variability must be accounted for, thus requiring different emulation strategies, such as heteroscedastic GPs [84].

### (b) Forward UQ

Forward UQ methods propagate *prior* input uncertainties through the computer model to assess their effect on the model outputs. Predictions paired with uncertainties give meaningful predictive capabilities and help with robust decision-making. Emulators provide a fast and efficient way to explore model responses under different input scenarios, enabling forward UQ at a lower computational cost. The two main methods of forward UQ (as shown in figure 1) are uncertainty analysis and sensitivity analysis (SA) [85]. The former propagates uncertainty from various sources through the model to compute the model’s output uncertainty distribution. This output uncertainty, often measured by its variance, can then be decomposed to evaluate the influence of each input on the model’s output, which is SA [75,86].

The main goal of uncertainty analysis is to quantify the information lost owing to uncertainties in model inputs, parameters and assumptions[]. This is typically done using Monte Carlo techniques, where input parameters are treated to be random variables with known distributions. Large samples of input parameters are drawn, and the corresponding outputs are evaluated to produce the uncertainty distribution. When using emulation, the added uncertainty from using the emulator must be included in the analysis.

SA quantifies how the model inputs contribute to the output uncertainty, allowing input parameters to be ranked according to their influence [87,88]. There are two main approaches: global SA, which examines input–output relationships across the entire input space, and local SA, which focuses on small perturbations around a specific point [14]. Local SA is limited to small regions, typically using either automatic differentiation or derivative-based sensitivities [14,89], making it less suitable for nonlinear models. Global SA methods consider the effects of all inputs and their interactions, offering a comprehensive overview of their influence on the model output. However, this approach can be computationally expensive, requiring sufficient sampling of the input space to capture variability and interaction effects. Common global SA methods include variance-based approaches such as Sobol’ indices [90], Fourier amplitude sensitivity tests [91], Morris screening [14] and Shapley indices [92], which aim to calculate contributions from each input and their interactions on the output uncertainty. Each methodology has its own assumptions (e.g. parameter independence), which subsequently affect the reliability of the results. Emulators can also be used to perform SA more efficiently [93].

### (c) Inverse UQ

Inverse UQ focuses on quantifying the *posterior* uncertainty in model inputs, and the associated posterior uncertainty in model outputs. Part of inverse UQ is parameter estimation, which connects models to reality using observational data and predicts future states and outcomes of the system. Note that output uncertainty propagation based on prior input uncertainties can provide substantially different results than output uncertainty propagation based on posterior input uncertainties (i.e. after model calibration from data) [14].

In some cases, modellers adjust parameter values inside the model manually [94], though this can be very time-consuming and unreproducible. Alternatively, a distance function between simulations and observations can be specified, such as least squares, and minimized, providing a deterministic, single best set of parameter values [14,95]. These optimization problems can be ill-posed [96], i.e. with multiple local optima. In addition, optimization over all observations can lead to overfitting (overtuning) [95], where the model performs well for those outputs but exhibits unphysical behaviour in others.

To address these concerns as well as to perform a realistic assessment of parameter uncertainty in mathematical models, inverse UQ methods can be employed. Optimization methods can be combined with an appropriate observation error model (defined in equation (3.1)) to reformulate the least-squares problem into a frequentist statistical inverse problem [14]. This enables the calculation of parameter confidence intervals after estimation, similar to standard methods used in linear and nonlinear regression. In contrast [96], developed the Bayesian approach to inverse problems, which represents the observation $z_{i}$ as


(3.1) $$z_{i}=f({\text{x}}^{*})+e_{i},\;i=1,\ldots ,m,$$

where $e_{i}$ is a zero-mean random variable that corresponds to the observational noise. A prior distribution for model parameters π(x) is specified, and observations $z_{i}$ are used to derive the posterior distribution. This method provides parameter posterior uncertainty and considers the observation error (measurement uncertainty) as part of the analysis. However, failing to recognize that the mathematical model is not a perfect representation of the true process of interest can lead to biased and over-confident parameter estimates and predictions [97]. Similar to the study in [96], Kennedy & O’Hagan [98] assumed the existence of the ‘best’ input parameter setting ${\text{x}}^{*}$ and proposed to define a model discrepancy as the difference between the physical process and the mathematical model representation, and include it in the observation equation, i.e.


(3.2) $$z_{i}=f({\text{x}}^{*})+\delta_{i}+e_{i},\;i=1,\ldots ,m,$$

where the model discrepancy term δ is a zero-mean GP. This turns it into a problem where we need to infer parameters and model discrepancy jointly. However, this approach may suffer from non-identifiability issues that can be resolved by informative priors specification on the parameters and model discrepancy [97,99]. A frequentist approach to identifying model discrepancy is presented in [100]. We consider observation error and model discrepancy as part of our proposed UQ pipeline in figure 1.

Obtaining improved prior specifications can be challenging in most applications. In addition, solving a joint parameter and model discrepancy inference problem can be computationally infeasible in a high-dimensional space [80,101]. Instead, history matching (HM) can be used to obtain the regions of parameter space corresponding to a good match between observations and model representation [102]. Bayesian HM uses a distance function, commonly known as the implausibility measure, to iteratively rule out the implausible regions of parameter space. This implausibility measure only requires expectations and covariance structures specifications—namely, expectation and variance of the model output, variance of the observation error and model discrepancy variance—aligning with a Bayes Linear philosophy [103]. In practical applications, it is common to treat the model discrepancy as the modellers’ tolerance to model error (see [3] for more details). We also note that HM can be performed as a pre-calibration step [104].

## 4. Current trends, gaps, and opportunities for UQ in biological and healthcare domains

The application of UQ in biological and healthcare models is understudied and suffers from several critical knowledge gaps. To successfully adopt computational models in biological or clinical decision support systems, several challenges need to be overcome, as discussed below.

### (a) Incomplete model credibility assessment

While VV&UQ is prominent in engineering and biology, UQ is typically lacking. The clinical community shows skepticism in using mechanistic models as assistance tools in patient-specific evidence-based diagnosis, risk assessment and treatment decision support owing to models being simplifications of the complex human physiology. Biological and physiological mechanistic models have relatively fewer concrete ‘laws’ describing processes than traditional physical science domains [14]. For instance, the Navier–Stokes equation is a principal-based equation for fluid flow, yet its application to blood flow is non-trivial [105]. Standard epidemiological and ecological models, e.g. susceptible-recovered (SIR)-type models, come with their own limitations in mechanistically describing disease transmission [57].

**Opportunity:** To generate trust between researchers, scientists, clinicians and patients, a thorough assessment of model credibility (VV&UQ) is necessary [46,106–108]. Model credibility has been addressed in detail by several authors, including Erdemir *et al*. [109]. Clearly defining the context of use for a model, performing VV&UQ using appropriate output quantities and listing limitations of the model explicitly are a few of these important rules for credibility.

### (b) Lack of UQ understanding and incomplete UQ

We require a deeper understanding of UQ, which is the least understood component in the model credibility process. More specifically, the application of UQ to healthcare and biological systems is under-developed [46,54,107]. There is an urgent need for a synergy between experts in the UQ field and mathematical modellers, who are often underexposed to UQ tools.

In particular, *model discrepancy* between the mechanistic model and the real system is an often neglected source of uncertainty in healthcare and biological modelling studies, consequently leading to distorted predictions and biased uncertainty [12,97,110]. Although some studies have included model discrepancy in the UQ analysis [12,110–112], it remains a difficult problem to tackle owing to confounding interactions between model discrepancy and error parameters (see §3c) stemming from insufficient data or knowledge about the system [97,99]. This is a prevalent issue in biological problems, owing to missing physics or simplifying assumptions in the modelling framework, or numerical approximations in the numerical scheme used to solve the mechanistic model equations [99,113].

In addition, a complete picture of UQ is usually missing in biological and healthcare modelling research, leading to misestimated uncertainty. More specifically, failing to account for both types of UQ (i.e. forward and inverse) [114,115] can cause uncertainty misestimation. Furthermore, failing to jointly incorporate all inverse UQ sources, e.g. uncertainty in model parameters, measurements, numerical errors from discretization (e.g. partial differential equations (PDEs)) or model error, will cause incomplete UQ. While several studies in the literature [7,42,116] include uncertainty in model parameters, the effects of parameter fixing in the presence of a large number of parameters are an ongoing challenge.

**Figure 2.**
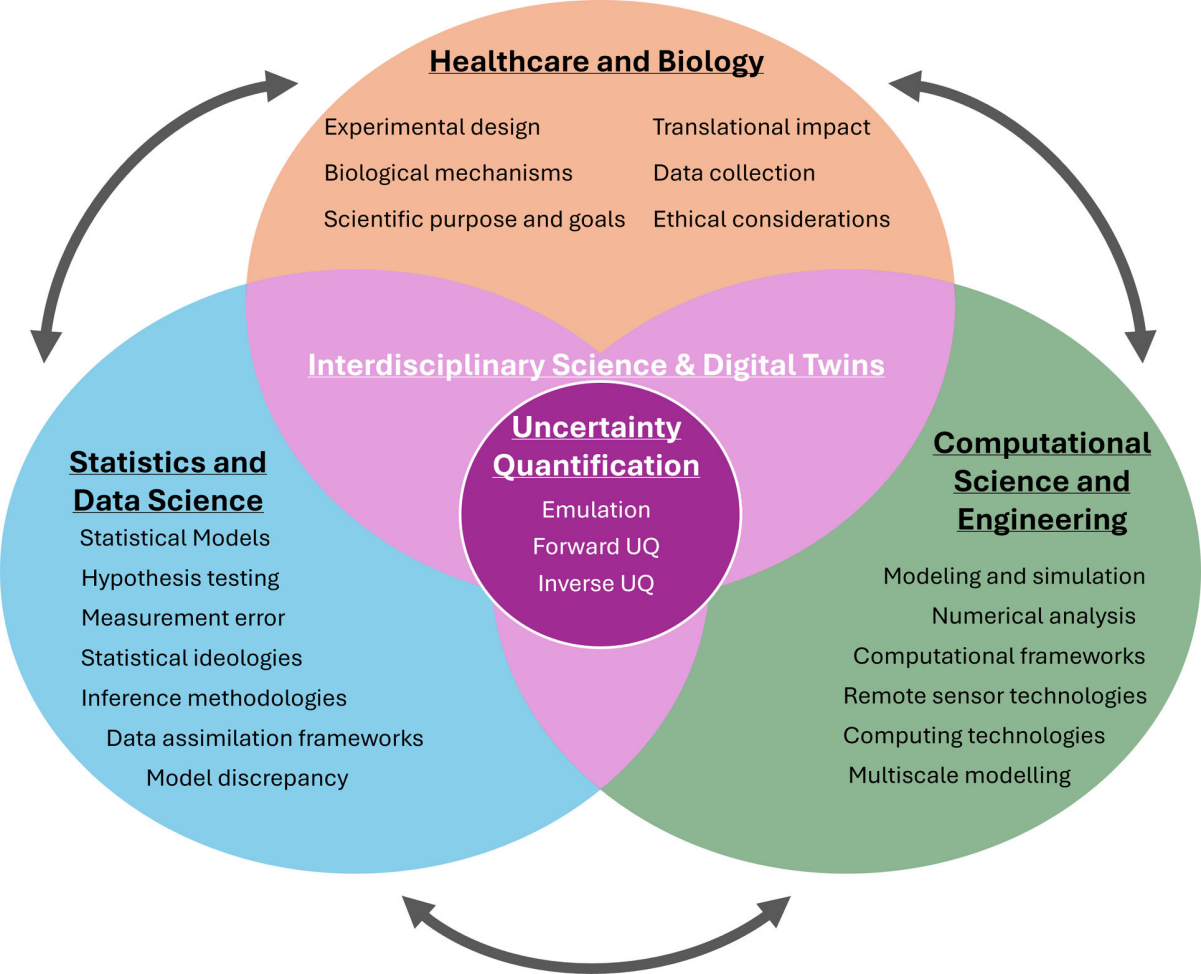
Overarching flow of expertise for UQ. Each subdomain has its own specific skill sets which, when applied together in an interdisciplinary setting, can be used to better explore uncertainty in biological and healthcare problems.

**Opportunity:** A holistic UQ approach will increase modelling rigour and reliability and generate confidence in them to guide healthcare decisions. Though forward UQ is useful for understanding predictive variability, inverse UQ provides information about posterior uncertainty and is crucial in determining confidence in model predictions in light of data. Coupling forward and inverse UQ can help address the issue of parameter identifiability, where mathematicians can work jointly with statisticians to integrate physiological prior knowledge into the statistical analysis [117]. Identifiability will be key as digital twins and their parameters are used as new disease biomarkers [14,15]. Team-based science that includes experts in mathematical modelling, computational statistics, computer science and biology (or physiology) is necessary for synergistic activities in digital twin development [17]. When addressing model discrepancy, modellers should collaborate with statisticians to identify specific components of the model that lack fidelity and provide prior evidence for discrepancy terms [11]. In addition, more investments should be made in educational literature [75,118] and workshops. We also emphasize, in contrast to prior perspective pieces (and reflected in figure 2), that **statisticians should be consulted and included in the team-science approach within biological and healthcare modelling.**

### (c) Adaptation of existing UQ methods

Biological and physiological models have complex, multi-scale mechanisms with limited, noisy observations obtained from experimental measurements [119,120]. Many biological inverse problems are ill-posed owing to non-identifiability between initial conditions, boundary conditions (BCs) and biological parameters, leading to multiple solutions of the system equations [119,120]. Thus, existing UQ methods require adaptation for healthcare and biological systems. As an example, SA for deterministic systems is commonly employed [114,121], yet biological agent-based modelling (e.g. cancer modelling) [5,122] requires innovative SA methods to accommodate the stochastic output. Biological and healthcare models typically have multiple internal states, yet typical SA quantifies parameter effects on a single quantity of interest.

**Opportunity:** Biological and healthcare models are distinct in their complexity and underlying mechanisms. New mathematical and statistical innovations to handle multi-output quantities of interest and quantify correlations/covariance of the model outputs are warranted. In addition, there is a critical need for determining parameter identifiability [123,124] in complex, biological systems. UQ in systems described by stochastic differential equations [125] requires innovations to deal with the problem of intractability of the data likelihood. For instance, the development of accurate and efficient likelihood-free approaches (e.g. approximate Bayesian computation, ABC[126]) based on data features lends itself to the opportunity of devising an algorithm to extract informative and low-dimensional features from the data, and to appropriately weight and combine them.

### (d) Inconsistent UQ language

Although UQ is adopted in multiple disciplines, the language used differs. For interdisciplinary research, it is important that experts in mathematics, statistics, engineering, healthcare and biology develop a common language. For example, an approximate mathematical model is referred to as a ‘metamodel’, ‘surrogate model’ or ‘emulator’ in different disciplines [110]. In a similar way, ‘calibration’ may imply hand-tuning or manual parameter adjustments, whereas formal model calibration in statistical (and now also engineering) applications requires numerical procedures, measurement error models and transparent assumptions about the model and data [12,14]. The term ‘model’ in engineering typically implies a mechanistic model, while in statistics, it implies a statistical model that may have different (free or fixed) parameters.

**Opportunity:** Scientists will benefit from an understanding of domain-specific UQ language. Developing collaborations across fields (see figure 2) helps alleviate potential misunderstandings in notation and language. We recommend that scientists read UQ articles from other domains, consider UQ texts that clearly communicate different definitions and languages [75,118], and take time to interact with different scientific domains on UQ. This will drive new research directions where UQ is at the intersection of multiple fields.

### (e) Ambiguity about the right model complexity

Determining the correct model complexity for a specific problem is non-trivial and, in UQ, applies to both the mechanistic model and statistical model complexity. The former includes choosing model fidelity (e.g. the inclusion or exclusion of certain biological processes) [8,127], or the choice of ODE versus PDE models [14]. This choice also affects the degree and form of model discrepancy [12]. Keeling and Eames [128] illustrate how epidemiological model fidelity affects simulations of disease infection. In addition, the parameter complexity (i.e., number of inferred parameters) of the model can lead to differences in the overall conclusions of a study. Colunga *et al*. [89] showed that differences in the inferred parameter subset led to different conclusions regarding heart-failure outcomes. The choice of statistical error model and its complexity (e.g., independent and identically distributed versus correlated errors) may affect the estimated uncertainty [110].

**Opportunity:** An alternative to choosing one fidelity of the mechanistic model is combining models of different fidelities or scales (e.g. from molecule to cell, tissue and last organ level) in a multi-fidelity, multi-scale modelling framework [14,109,119]. Systems with poorly understood physics or limited data using multi-fidelity modelling require new innovations in UQ methods as well [119]. While multiple papers use SA for parameter fixing *a priori* [12,89,107], relatively few papers use statistics-driven criteria, such as information criteria [12,129], for identifying model complexity. Again, we emphasize that computational modellers and statisticians should work together to identify model complexity in both the infrastructure of the mechanistic and statistical model when conducting inverse UQ.

### (f) Difficulties with data

Limited, incomplete and noisy data are a common issue in modelling healthcare and biological systems [119]. Many biological and healthcare research use averaged or non-dynamic (e.g. blood pressure) data [14]. However, mechanistic models may predict non-averaged or dynamic outputs [12] and would benefit from higher-fidelity data sources. Other factors include inadequate experimental designs for model calibration, incomplete health history or noisy measurement devices [14]. These factors result in model parameters that are practically non-identifiable when solving statistical inference problems [14,124]. Data bias is another issue. Under-representation of individuals in the data for model training will lead to biased, suboptimal healthcare for the particular group [15]. The guidelines introduced by [14] emphasize data bias in some cardiovascular modelling studies owing to the absence of factors such as sex from the experimental study. This has resulted in biased cardiovascular models that are male specific, limiting their application to female patients and biasing future UQ on these models.

Dealing with high-dimensional and multi-modality data is another challenge in biomedical research. Large and complex ‘omics’ datasets as well as electronic health records and medical imaging data are usually heterogeneous, making a proper UQ study a challenging task [130,131]. Analysing medical images, such as segmenting and meshing the heart for simulation [132], relies on manual interventions by trained experts, rendering clinical digital twins currently infeasible.

**Opportunity:** Numerous initiatives aim to collect an abundance of data for ML and AI. However, **a major question is whether these data are accurate or informative for modelling and simulation**. Putting UQ at the forefront will enable clear communication about which data are appropriate for a given problem. Data and measurement devices are imperfect, and accounting for their limitations in a statistical framework will ensure that results and conclusions from the data are reliable and credible. Modellers and statisticians should work in concert to identify beneficial data for research projects and collaborate with domain experts and experimentalists to build new experimental designs (see figure 2). Efforts to ensure that computational modelling enhances healthcare for all patients, regardless of their demographics and especially in the cases when data are limited, are needed. Integrating physical constraints such as conservation, invariance or symmetry into the AI- and ML-based analysis may be beneficial.

### (g) Emulators and UQ

Emulation methods, while powerful, have shortcomings. Their approximation of the simulator introduces an extra layer of uncertainty into the analysis. Conducting UQ analysis within an emulation framework poses additional challenges owing to accuracy loss, and the development of emulation UQ methods that are able to achieve accurate results is an active area of research [133,134]. Recently, there has been a surge in studies adopting physics-informed emulation in place of a data-driven approach based on numerical simulation data [64,66,67]. For instance, the study by Grandits *et al*. [64] uses a graph neural network for emulating a cardiac mechanics model. Physics-informed emulation has more often been employed from a purely predictive perspective [64,66,67] rather than from an inference perspective. An example from the latter is the study by Ahmadi Daryakenari *et al*. [135] that employs a physics-informed neural network for parameter estimation and missing physics identification in systems biology.

**Opportunity:**Mathematicians, engineers and statisticians all employ different emulation strategies (e.g. GPs, polynomial chaos expansions, neural networks, etc.) [64,119]. Clear communication about emulation performance metrics and their ability to solve forward and inverse problems is warranted. In particular, understanding which emulation strategies return the most accurate predictions and investigating how predictive accuracy correlates with inference accuracy is needed. In biological mechanistic modelling, substantial efforts are required to integrate physics-based emulation within a multi-fidelity, multi-scale and multi-physics framework [136]. We emphasize that integrating physics with data provides a possible solution to the challenge of contemporary AI by making ‘black-box’ (data-driven) ML methods ‘explainable’ and advocate for educating and encouraging non-computational scientists to use mechanistic modelling in a synergistic fashion with AI and ML.

### (h) Incorporating patient-specific healthcare into a population framework

Extending predictive models to a general patient cohort is non-trivial [15]. First, mechanistic models must be extended to reflect physiological and demographic heterogeneity of the population (e.g. biologically female and male patients) to avoid model bias in clinical decision-making. Studies so far have only dealt with relatively small patient groups (approx. 100 patients) [137], and hence substantial efforts will be required for larger scalability. Second, AI and ML method extensions are lacking, more specifically building emulators that account for anatomical variability for a population, or conducting the UQ analysis in a population framework using a Bayesian hierarchical model. Integrating population data in a dual mechanistic-statistical framework will require significant innovations in inverse UQ, as measurement noise and parameter influence may be patient-specific, making it difficult to expand to a population.

**Opportunity:** Statisticians have experience in handling both individual and population-level data (e.g. Bayesian hierarchical models) and should be consulted in the development of mechanistic models at the population level. Population data may have distinct characteristics to patient-specific data owing to issues with data collection strategies at large scales, possibly resulting in missing or incomplete data, and even outliers. To tackle this, the available data may be supplemented with synthetic cases obtained through computational simulations. In addition, reliable data-sharing platforms will play an important role in providing additional datasets [15]. Epidemiology modellers routinely simulate large-scale dynamics using population data and will be a valuable contributor to statisticians and healthcare modellers to better translate patient-specific to population simulation.

### (i) Ethical and legal considerations

Before the incorporation of digital twins and AI in a clinical practice, several critical ethical considerations need addressing, such as (i) patient consent for their medical data to be used, (ii) data privacy and protection, and (iii) transparency and accountability [138,139]. In the context of extending the UQ framework from a patient to a population level, where access to data-sharing platforms is important, consent and data sharing are not straightforward to implement in a clinical pipeline. For instance, it has been argued that access to shared patient data should be granted to national and international bodies under certain conditions, such as securing and de-identifying data (i.e. removing personal information, such as name, address and social security numbers) [140]. There is also ambiguity about transparency and accountability when using black-box algorithms for UQ. For example, how transparent should a clinical professional be about their limited understanding of how these algorithms work to guide the medical decision support, how will this relate to the *legal* ‘right to explanation’ under the EU General Data Protection Regulation [106,139], and who will be held accountable if anything goes wrong? These concerns could be partially alleviated by designing explainable AI methodologies that enable UQ.

**Opportunity:** Science will only have its effect on everyday life when it is clearly communicated and disseminated through appropriate policy experts. In light of the COVID-19 pandemic and in preparation of the era of digital twins, modellers, statisticians and healthcare experts must communicate ideas in a digestible manner. Moreover, UQ must be incorporated in the future to emphasize the possible outcomes and precision of predictions or conclusions from computational frameworks. The use of modelling without proper UQ can over-promise the potential of computational science. As argued throughout this text, UQ and the concept of uncertainty must be (i) integrated within every problem that uses computational modelling, and (ii) clearly explained for multiple audiences to ensure that credibility and trust are at the forefront of a given application area.

## 5. Conclusions and articles in the special issue-Part 1

Computational modelling and simulation of healthcare and biological systems is a rapidly evolving area, with numerous opportunities to affect policy decisions, healthcare practices and new technologies. Like physics, climate science and aerospace, the results in healthcare and biological applications should be scrutinized via a proper quantification of uncertainty. In this review article, we emphasize the need for synergy between experts in statistics, modelling and simulation, and domain science. This volume of the two-part special issue contains articles that address multiple challenges discussed in §4.

We begin with the opinion article by Tsaneva-Atanasova *et al.* [141], which examines in detail the role that UQ plays in informing clinical decision-making processes. The authors discuss the crucial role of understanding and managing the uncertainties present in clinical data (such as measurement error), diagnostic tools and treatment outcomes. The authors demonstrate how effectively addressing and decoding uncertainties could significantly enhance the accuracy and robustness of clinical decisions, ultimately leading to better patient outcomes and more informed healthcare practices.

Scientific ML holds significant value, especially in expediting simulation and UQ. For instance, the study by Böttcher *et al*. [142] aims to improve existing control and optimization techniques specifically for human biological models where typical physics-based approaches are not suitable. They use dynamics-informed artificial neural network (ANN) controllers as an alternative approach to using medical digital twins, focusing on the control of agent-based models in biomedical applications. The authors show that the ANN approach is robust and computationally efficient, allowing for the calculation of parametric uncertainty. In addition, the study by De Florio *et al*. [143] provides a novel strategy for handling ‘total uncertainty’, including epistemic, aleatoric and model-form uncertainty within the framework of physics-informed ML. Their methodology, applied to a reduced-order computational haemodynamics model, showcases the importance of model misspecification in the emulator training process.

In the field of emulation, the study by Paun *et al*. [144] compares GPs and polynomial chaos expansions as emulators for forward and inverse problems in the context of pulmonary blood flow modelling. Their results indicate that GPs outperform slightly, but consistently polynomial chaos expansions across every comparison, and that a similar performance is obtained for the emulators of multivariate output and reduced output from the fluid-dynamics models. The study by Salter *et al.* [145] provides a novel emulation approach to overcome issues in population dynamics via a Poisson log-normal principal component analysis emulation strategy applied to a COVID-19 model. The authors show that the emulator provides predictions that capture complex correlations between the true model outputs.

In the study by Bajaj *et al*. [146], the authors develop a new Bayesian approach to account for the dynamics of the reproduction number, $R_{t}$, in epidemiological models. Their approach highlights the importance of $R_{t}$ history and provides a method for quantifying uncertainty in the time-dependent values of $R_{t}$. In addition, the use of novel Bayesian approaches using computationally cheaper surrogate models is presented in the study by Richter *et al*. [147], which demonstrates how to efficiently quantify uncertainties in three-dimensional cardiovascular fluid-dynamics simulations using clinical measurements. The authors only evaluate the three-dimensional model once for an initial choice of boundary conditions and use the result to create a highly accurate zero-dimensional (0D) surrogate. The authors then perform sequential Monte Carlo using the optimized 0D model to derive the high-dimensional (Windkessel) boundary conditions posterior distribution, thus significantly reducing the computational complexity of the problem.

The study by Dadashova *et al*. [148] develops a method for determining identifiable parameters in the physiologically based pharmacokinetic brain model that comprises coupled systems of ODEs and a large number of parameters. These models are crucial for understanding the movement of drugs and other chemical compounds within the body. The authors propose to combine the parameter subset selection method with Bayesian inference to obtain a subset of identifiable parameters with quantified uncertainties.

In the field of cardiac electrophysiology, Del Core & Mirams [149] consider the stochastic nature of ion-channel behaviour. The study presents a state-space model based on stochastic reaction networks, which retain statistical properties that can be used to identify parameters in the system. The authors show that the proposed method outperforms standard inference in ion-channel models and is able to detect distinct components of measurement error that are typically missed in deterministic-based inference. The study by Shuttleworth *et al*. [150] calibrates a model with a voltage-sensitive potassium ion-channel current to newly collected data from patch-clamp experiments. The authors assess how each model predicts the dynamics of the patch-clamp protocol and report variability in parameter estimates with each protocol. The authors show that heterogeneity across experimental designs and cell types accounts for a missing latent effect within the modelling framework, and that incorporating this missing effect may benefit the applicability of these models to a wider set of experiments. Furthermore, Patten-Elliott *et al*. [151] focus on defining the mechanisms by which drug compounds bind to the human Ether-a-go-go Related Gene to reduce uncertainty when assessing the risk of cardiac arrhythmia. They present automated optimal experimental design techniques to design voltage-clamp protocols that can be used in voltage-clamp experiments to better distinguish between different models of the drug-binding mechanism.

We end the special issue with a study by Rabbani *et al*. [152] that develops an adaptive three-dimensional image synthesis technique to reconstruct the inhomogeneous, porous microstructure of the meniscus, a fibrous knee tissue. These reconstructions are used to model hydraulic permeation and mechanical testing. By analysing 1500 synthesized geometries, they introduce a feature extraction technique, allowing for the systematic generation of perturbed three-dimensional biomaterial models. This framework helps identify structure–function relationships, offering insights into the connection between macroscopic biophysical properties and microscale tissue characteristics.

## Data Availability

This article has no additional data.
